# Impact of High-Sensitivity C-Reactive Protein Cutoff Selection on Cardiovascular Risk Classification Beyond Lipid Measurements in Korean Adults: A KNHANES 2024 Study

**DOI:** 10.3390/metabo16080531

**Published:** 2026-07-27

**Authors:** Rihwa Choi, Gayoung Chun, Sang Gon Lee, Sung-Eun Cho

**Affiliations:** 1 Laboratory Medicine Center, GC Labs, Yongin 16924, Republic of Korea; 2 Department of Laboratory Medicine and Genetics, School of Medicine, Sungkyunkwan University, Seoul 06351, Republic of Korea; 3 Center for Global Health & Infectious Disease Research, GC Labs, Yongin 16924, Republic of Korea; 4 Endocrine Substance Analysis Center, GC Labs, Yongin 16924, Republic of Korea

**Keywords:** high-sensitivity C-reactive protein, cardiovascular risk, risk reclassification, Korea National Health and Nutrition Examination Survey

## Abstract

**Background/Objectives**: This study aimed to describe the distribution of high-sensitivity C-reactive protein (hsCRP) in Korean adults and to evaluate the incremental detection of elevated hsCRP beyond lipid abnormalities using nationally representative data. **Methods**: We analyzed 2024 Korean National Health and Nutrition Examination Survey data from adults aged ≥20 years with available hsCRP and lipid measurements, excluding pregnant women. Elevated hsCRP was defined using three cutoffs: ≥1 mg/L, ≥2 mg/L, and >3 mg/L. Additional detection by hsCRP was defined as the proportion of participants without any lipid abnormality, as defined according to the NCEP ATP III criteria, who had elevated hsCRP. **Results**: A total of 5769 adults were included in the common hsCRP and lipid analysis population. In the survey-weighted analysis, 72.5% of participants had hsCRP < 1 mg/L, whereas 12.8%, 6.2%, 6.9%, and 1.6% had hsCRP 1 to <2, 2 to ≤3, >3 to <10, and ≥10 mg/L, respectively. The proportions of participants with hsCRP ≥1 mg/L, ≥2 mg/L, and >3 mg/L were 27.5%, 14.7%, and 8.5%, respectively. Any lipid abnormality was present in 34.4% of participants. Among 3861 participants with normal lipid profiles, elevated hsCRP was found in 22.6%, 11.7%, and 6.8%, corresponding to overall additional detection rates of 14.8%, 7.7%, and 4.4% at cutoffs of ≥1, ≥2, and >3 mg/L, respectively. **Conclusions**: hsCRP identified additional individuals who were not detected by conventional lipid abnormalities. These findings provide nationally representative descriptive data on hsCRP distribution and threshold-based classification in Korean adults and underscore the need for population-specific interpretation of hsCRP cutoffs.

## 1. Introduction

Cardiovascular disease remains a major public health burden worldwide, and lipid measurements are central to cardiovascular risk assessment and preventive decision-making. Total cholesterol, low-density lipoprotein cholesterol (LDL-C), high-density lipoprotein cholesterol (HDL-C), and triglycerides are routinely used to identify individuals at increased cardiometabolic risk [1,2,3,4]. However, atherosclerosis is not solely a lipid-driven process. Chronic low-grade inflammation also contributes to atherogenesis, plaque progression, and cardiovascular events [4,5]. Therefore, lipid-based assessment alone may not fully capture inflammatory residual risk, particularly among individuals who do not meet conventional lipid abnormality criteria [1,2,3,4,5,6,7].

High-sensitivity C-reactive protein (hsCRP) is the most widely studied circulating biomarker of low-grade systemic inflammation in cardiovascular risk assessment [1,2,3,4]. hsCRP reflects low-grade systemic inflammation that contributes to atherosclerotic plaque development, progression, and destabilization, thereby linking inflammatory metabolism to cardiovascular events [1,2,3,4,5,6,7]. Previous clinical and epidemiologic studies have shown that elevated hsCRP is associated with future cardiovascular events, even among individuals without marked lipid abnormalities [6,7,8,9,10,11]. Accordingly, hsCRP has been incorporated into cardiovascular prevention frameworks as an adjunctive marker that may refine risk assessment when treatment decisions are uncertain [1,4,7].

Two major hsCRP interpretation frameworks are commonly used. The traditional US Centers for Disease Control and Prevention (CDC) and American Hospital Association (AHA) categories classify hsCRP concentrations of <1.0, 1.0–3.0, and >3.0 mg/L as low, average, and high relative cardiovascular risk, respectively [1,2,3,4]. The conventional hsCRP categories of <1, 1–3, and >3 mg/L were originally proposed by the AHA and CDC based primarily on approximate tertile distributions observed in large adult populations, together with evidence of a graded association between hsCRP concentrations and subsequent cardiovascular risk [5,6]. These thresholds were, therefore, not derived from quintiles or from a Korean population-specific percentile distribution. In contrast, contemporary cholesterol management guidelines use hsCRP ≥ 2.0 mg/L as a risk-enhancing factor [1,2,3,4]. In particular, individuals with hsCRP values between 2.0 and 3.0 mg/L would be identified by the ≥2.0 mg/L criterion but would not be classified into the traditional >3.0 mg/L high-risk category. Therefore, the choice of hsCRP cutoff may substantially influence the number of individuals considered to have increased inflammatory risk.

This issue may be especially relevant in Asian populations [7,8,9]. The distribution of hsCRP differs by race and ethnicity, and East Asian populations have generally been reported to have lower hsCRP concentrations than several Western populations [7,8,9,10,11,12,13,14]. As a result, a high-risk threshold such as >3.0 mg/L may identify only a limited proportion of East Asian adults, whereas a lower threshold such as ≥2.0 mg/L may provide additional clinically meaningful information [7,10]. However, population-based evidence quantifying how many Korean adults would be newly classified as risk-positive by different hsCRP cutoffs, particularly among those without conventional lipid-based risk factors, remains limited [15]. Accordingly, hsCRP cutoffs are not specified in the Korean dyslipidemia guidelines [15].

The Korea National Health and Nutrition Examination Survey (KNHANES) is a nationally representative database that provides reliable information on the Korean general population, and the most recent data from 2024 have been released [16,17]. Therefore, the primary objective of this study was to identify additional individuals with elevated hsCRP at different cutoffs, particularly among those without lipid abnormalities. In addition, using the potential confounders available in KNHANES, we performed subgroup analyses to identify factors associated with elevated hsCRP at each cutoff.

## 2. Materials and Methods

### 2.1. Study Population

This study was a cross-sectional analysis of data from the 2024 KNHANES, conducted by the Korea Disease Control and Prevention Agency [16,17]. KNHANES is a nationwide health survey designed to produce representative estimates of health status, health behaviors, chronic disease prevalence, and nutritional status among the non-institutionalized Korean population [16,17,18].

The inclusion criteria for the primary analysis were as follows: (1) participation in the 2024 Korea National Health and Nutrition Examination Survey (KNHANES); (2) age ≥ 20 years; (3) non-pregnant status [17]; (4) availability of hsCRP measurements; and (5) availability of results for all four lipid tests, including total cholesterol, HDL-C, LDL-C, and triglycerides. The exclusion criteria were age < 20 years, pregnancy [17], missing hsCRP measurements, and missing data for one or more of the four lipid measurements.

After applying these criteria, the primary analysis population was used to describe baseline characteristics, estimate the survey-weighted prevalence of elevated hsCRP, and generate heatmaps. Subgroup analyses were then conducted within this population according to the purpose of each analysis.

Subgroup analysis 1 included participants in the primary analysis population with normal results for all four lipid tests, regardless of covariate completeness. This subgroup was used to estimate the survey-weighted prevalence of elevated hsCRP among participants without lipid abnormalities.

Subgroup analysis 2 included participants in the primary analysis population with complete covariate data for multivariable logistic regression. Complete covariate data were defined as non-missing information for all variables included in the models: age group, sex, smoking status, obesity, diabetes status, physical activity limitation, alcohol use, hypertension, physician-diagnosed dyslipidemia, history of cardiovascular disease, kidney disease, and the four lipid abnormality indicators—high total cholesterol, high LDL-C, high triglycerides, and low HDL-C. Participants with missing data for any of these covariates were excluded from subgroup analysis 2. This subgroup was used to identify factors independently associated with elevated hsCRP.

Because the primary aim was to estimate the additional proportion of the Korean general population identified by hsCRP testing beyond conventional lipid testing, participants with hsCRP concentrations ≥10 mg/L were included in the primary analysis. To assess the potential influence of acute or transient inflammatory conditions, we additionally performed a sensitivity analysis excluding participants with hsCRP concentrations ≥10 mg/L [5].

### 2.2. Analytical Methods

Detailed information on the laboratory methods, instruments, and reagents used in the KNHANES health examination survey is available on the official KNHANES website [16]. The lipid variables used in this study were total cholesterol, HDL-C, triglycerides, and direct LDL-C [16,17,19,20,21]. Total cholesterol, HDL-C, LDL-C, and triglycerides were directly measured using Roche reagents (CHOL2, HDLC4, LDLC3, and TRIGL, respectively) on a Cobas 8000 c702 analyzer (Roche Diagnostics GmbH, Mannheim, Germany) [16,17]. hsCRP was measured using a particle-enhanced immunoturbidimetric assay on a cobas 8000 analyzer with Cardiac C-Reactive Protein (Latex) High Sensitive reagent (Roche Diagnostics GmbH, Mannheim, Germany). The analytical reportable range for hsCRP was 0.2–300.0 mg/L [16,17]. hsCRP values reported as below the lower reportable limit, such as “<0.2 mg/L,” were coded as 0.199 mg/L for analysis [16,17]. This substitution did not affect risk classification according to the hsCRP thresholds used in this study, because the primary hsCRP cutoffs were 1.0, 2.0, and 3.0 mg/L [1,2,3,4].

### 2.3. Definitions

For cutoff-based analyses, elevated hsCRP was defined using three thresholds: hsCRP ≥ 1 mg/L, hsCRP ≥ 2 mg/L, and hsCRP > 3 mg/L. These cutoffs were derived from different guidelines and published studies [1,2,3,4,5,6]. Given that the primary objective of this study was to identify additional individuals with elevated hsCRP at different thresholds, we used a cutoff of >3 mg/L rather than categorizing hsCRP into predefined risk groups. Because these cutoff-based variables are cumulative and not mutually exclusive, they were analyzed separately rather than as components of a stacked distribution.

Lipid categories were defined according to the National Cholesterol Education Program Adult Treatment Panel III (NCEP ATP III) criteria [20,21]. Total cholesterol was categorized as desirable (<200 mg/dL), borderline high (200–239 mg/dL), or high (≥240 mg/dL). LDL-C was categorized as optimal (<100 mg/dL), near optimal/above optimal (100–129 mg/dL), borderline high (130–159 mg/dL), high (160–189 mg/dL), or very high (≥190 mg/dL). Triglycerides were categorized as normal (<150 mg/dL), borderline high (150–199 mg/dL), high (200–499 mg/dL), or very high (≥500 mg/dL). HDL-C was categorized as low (<40 mg/dL), intermediate (40–59 mg/dL), or high (≥60 mg/dL).

For the primary definitions of lipid abnormalities used in this study, high total cholesterol was defined as total cholesterol ≥ 240 mg/dL, high LDL-C as LDL-C ≥ 160 mg/dL, and high triglycerides as triglycerides ≥ 200 mg/dL. Low HDL-C was defined using sex-specific cutoffs of <40 mg/dL in men and <50 mg/dL in women. Participants were classified as having normal results for all four lipid tests if they had total cholesterol < 240 mg/dL, LDL-C < 160 mg/dL, triglycerides < 200 mg/dL, and HDL-C ≥ 40 mg/dL in men or ≥50 mg/dL in women. Participants with at least one of these abnormalities were classified as having any lipid abnormality.

Covariates included in the multivariable logistic regression models were age group, sex, smoking status, obesity, diabetes status, physical activity limitation, alcohol use, hypertension, physician-diagnosed dyslipidemia, history of cardiovascular disease, kidney disease, and the four lipid abnormality indicators.

Age was categorized into 10-year groups: 20–29, 30–39, 40–49, 50–59, 60–69, 70–79, and ≥80 years. Smoking status was defined using variables for cigarette smoking and heated tobacco use. Obesity was defined as a body mass index ≥ 25 kg/m^2^. Diabetes status was classified as normal, prediabetes, or diabetes according to KNHANES definitions [16,17]. Diabetes was defined as fasting plasma glucose ≥ 126 mg/dL, current use of glucose-lowering medication, insulin treatment, physician-diagnosed diabetes, or HbA1c ≥ 6.5%. Prediabetes was defined as fasting plasma glucose of 100–125 mg/dL or HbA1c of 5.7–6.4% among participants who did not meet the criteria for diabetes [16,17]. Normal glucose metabolism was defined as fasting plasma glucose < 100 mg/dL and HbA1c < 5.7% among participants who did not meet the criteria for diabetes or prediabetes [16,17]. Hypertension was defined as systolic blood pressure ≥ 140 mmHg, diastolic blood pressure ≥ 90 mmHg, current use of antihypertensive medication, or physician-diagnosed hypertension [16,17]. A history of cardiovascular disease was defined as a history of stroke, myocardial infarction, or angina. Kidney disease was defined as physician-diagnosed kidney disease or an estimated glomerular filtration rate < 60 mL/min/1.73 m^2^ [22,23]. The estimated glomerular filtration rate was calculated using the 2009 CKD-EPI equation, as recommended in the 2021 evidence-based guideline for chronic kidney disease in primary care published by the Korean Academy of Medical Sciences and the Korea Disease Control and Prevention Agency under the supervision of the Korean Society of Nephrology [22,23].

### 2.4. Statistical Analysis

All analyses accounted for the complex sampling design of KNHANES by incorporating sampling weights, stratification variables, and primary sampling units [16,17]. Continuous variables were summarized as survey-weighted means ± standard errors (SEs), except for hsCRP, which was summarized as a survey-weighted median and interquartile range because of its right-skewed distribution. Categorical variables were summarized as unweighted n/N and survey-weighted percentages with 95% confidence intervals. The survey-weighted prevalence of elevated hsCRP at cutoffs of ≥1, ≥2, and >3 mg/L was estimated overall and according to sex, age group, lipid category, and combined lipid status. Heatmaps were generated to visualize the weighted prevalence across these strata. Bar graphs were used to show the prevalence of elevated hsCRP in the lipid-normal subgroup, defined as participants with normal results for all four lipid tests. Survey-weighted multivariable logistic regression analyses were performed to identify factors associated with elevated hsCRP at cutoffs of ≥1, ≥2, and >3 mg/L. Adjusted odds ratios and 95% confidence intervals were estimated and presented in forest plots. Statistical analyses were performed using R software version 4.5.2 (R Foundation for Statistical Computing, Vienna, Austria). *p* value < 0.05 was considered statistically significant.

## 3. Results

### 3.1. Study Population Characteristics

**Figure 1:**
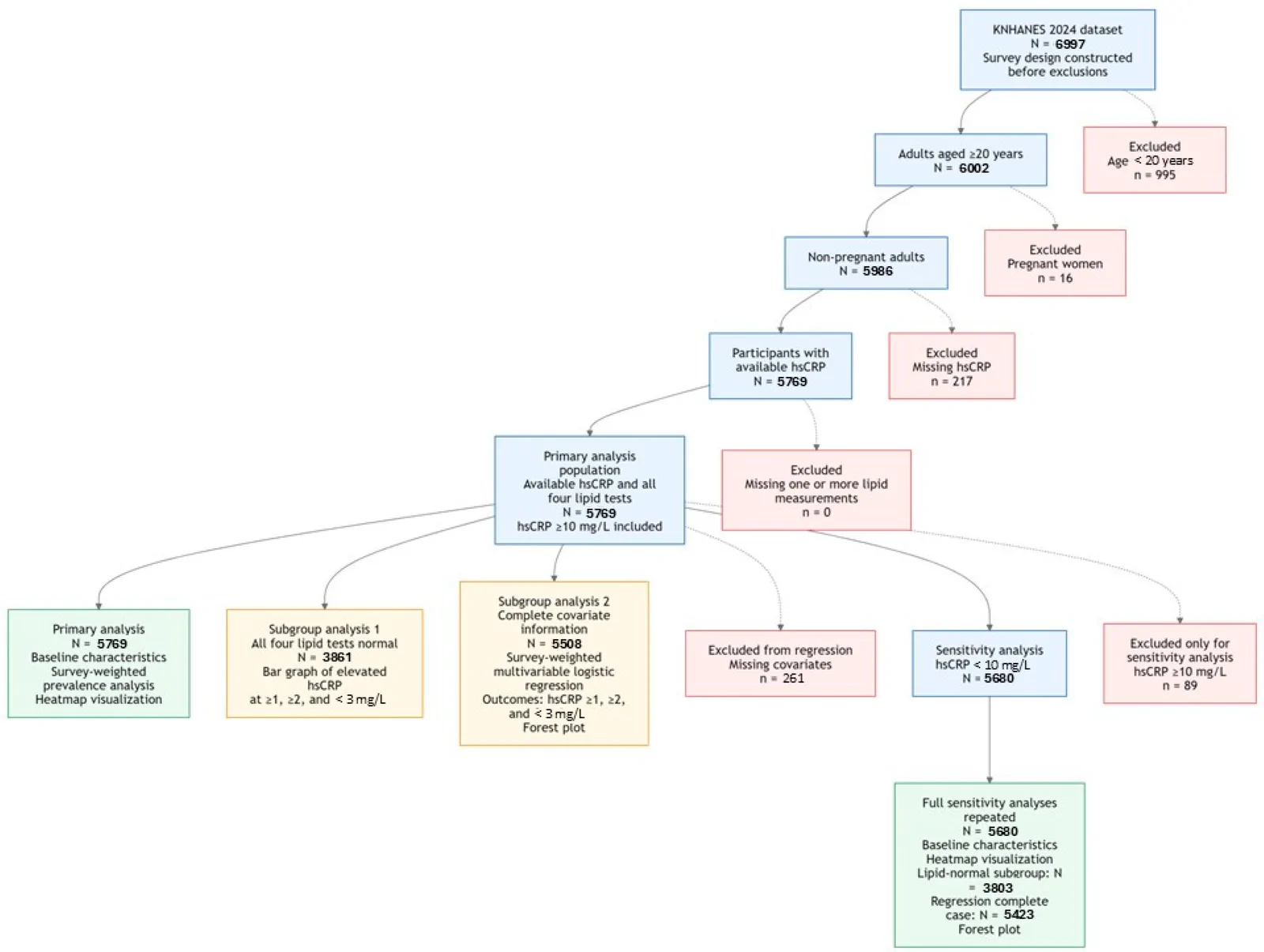
Flow diagram of the study population. After excluding participants aged < 20 years, pregnant women, and those with missing hsCRP measurements, 5769 adults with available hsCRP measurements and results for all four lipid tests were included in the primary analysis. Participants with hsCRP concentrations ≥ 10 mg/L were retained in the primary analysis. Subgroup analysis 1 included 3861 participants with normal results for all four lipid tests, whereas subgroup analysis 2 included 5508 participants with complete covariate data for multivariable logistic regression. For the sensitivity analysis, 89 participants with hsCRP concentrations ≥ 10 mg/L were excluded, leaving 5680 participants.

The study flow is shown in Figure 1. The 2024 KNHANES dataset included 6997 participants. After excluding 995 participants aged <20 years, 6002 adults aged ≥20 years remained. Of these, 16 pregnant women and 217 participants with missing hsCRP measurements were excluded. All remaining participants had available results for all four lipid tests, including total cholesterol, HDL-C, LDL-C, and triglycerides; therefore, no additional participants were excluded because of missing lipid measurements. The final primary analysis population comprised 5769 adults. Their baseline characteristics are presented in Table 1. The survey-weighted mean age was 50.0 years, and the survey-weighted proportion of men was 50.4%. Using the composite lipid abnormality definition, 34.4% of participants had at least one abnormal lipid parameter. Among the individual lipid components, low HDL-C defined by sex-specific criteria was the most common abnormality, observed in 17.8% of participants. Triglyceride levels ≥ 200 mg/dL and LDL-C levels ≥ 160 mg/dL were each observed in 12.8% of participants, whereas total cholesterol levels ≥ 240 mg/dL were observed in 10.6%.

**Figure 2:**
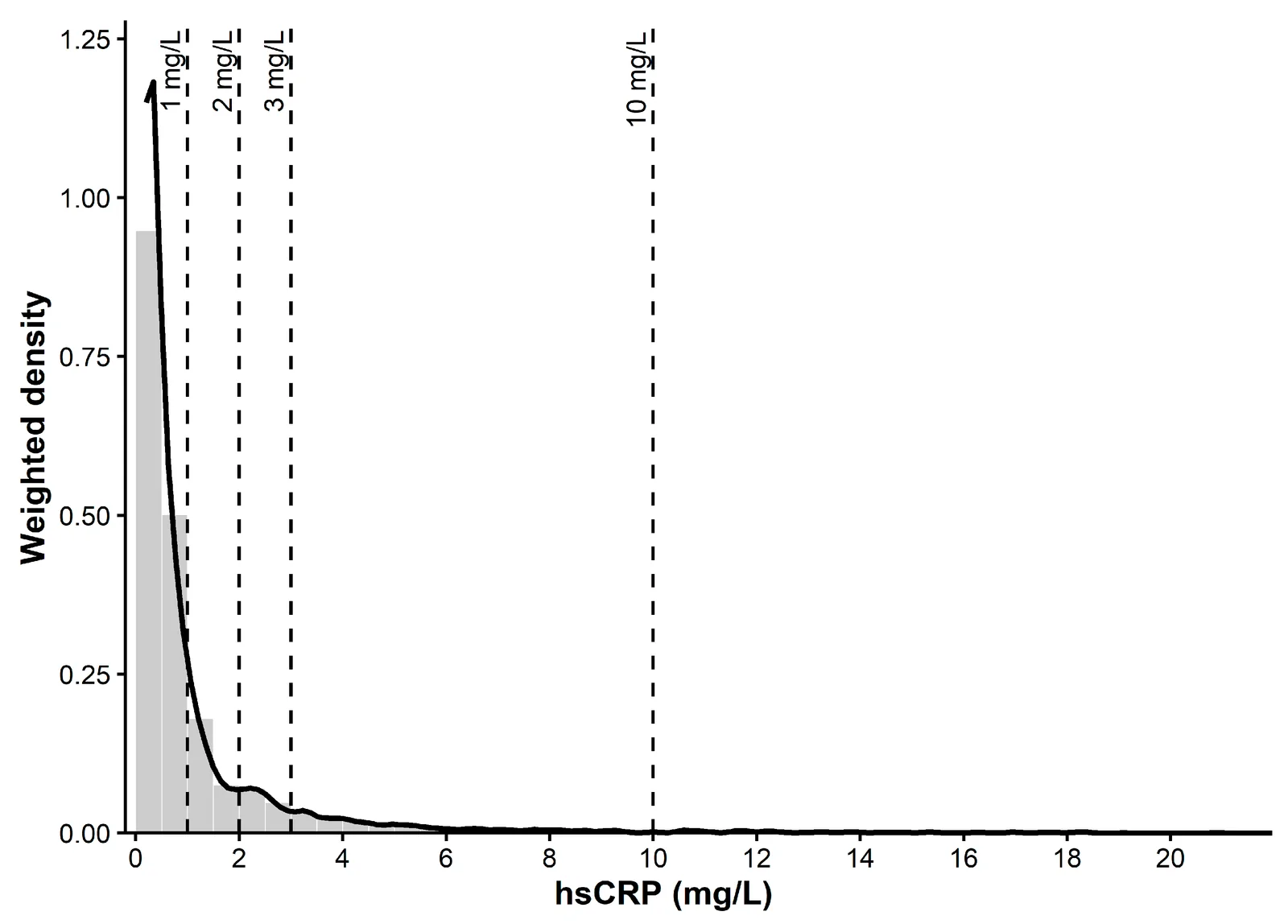
Distribution of high-sensitivity C-reactive protein (hsCRP) concentrations in Korean adults. The histogram and density curve show the survey-weighted distribution of hsCRP concentrations among non-pregnant Korean adults aged ≥ 20 years in the 2024 Korea National Health and Nutrition Examination Survey. Vertical dashed lines indicate hsCRP cutoffs of 1, 2, 3, and 10 mg/L.

In the survey-weighted analysis, 72.5% of 5769 participants had hsCRP < 1 mg/L. The proportions of participants with hsCRP 1 to <2 mg/L, 2 to ≤3 mg/L, >3 to <10 mg/L, and ≥10 mg/L were 12.8%, 6.2%, 6.9%, and 1.6%, respectively. When hsCRP was evaluated using cumulative cutoffs, 27.5% of participants had hsCRP ≥ 1 mg/L, 14.7% had hsCRP ≥ 2 mg/L, and 8.5% had hsCRP > 3 mg/L (Figure 1 and Figure 2).

The prevalence of elevated hsCRP differed by sex. Among men, the survey-weighted prevalence of hsCRP ≥1, ≥2, and >3 mg/L was 31.0%, 16.2%, and 9.9%, respectively. Among women, the corresponding prevalence was 24.0%, 13.2%, and 7.6%, respectively. Thus, men had a higher survey-weighted prevalence of elevated hsCRP at all three cutoffs.

**Figure 3:**
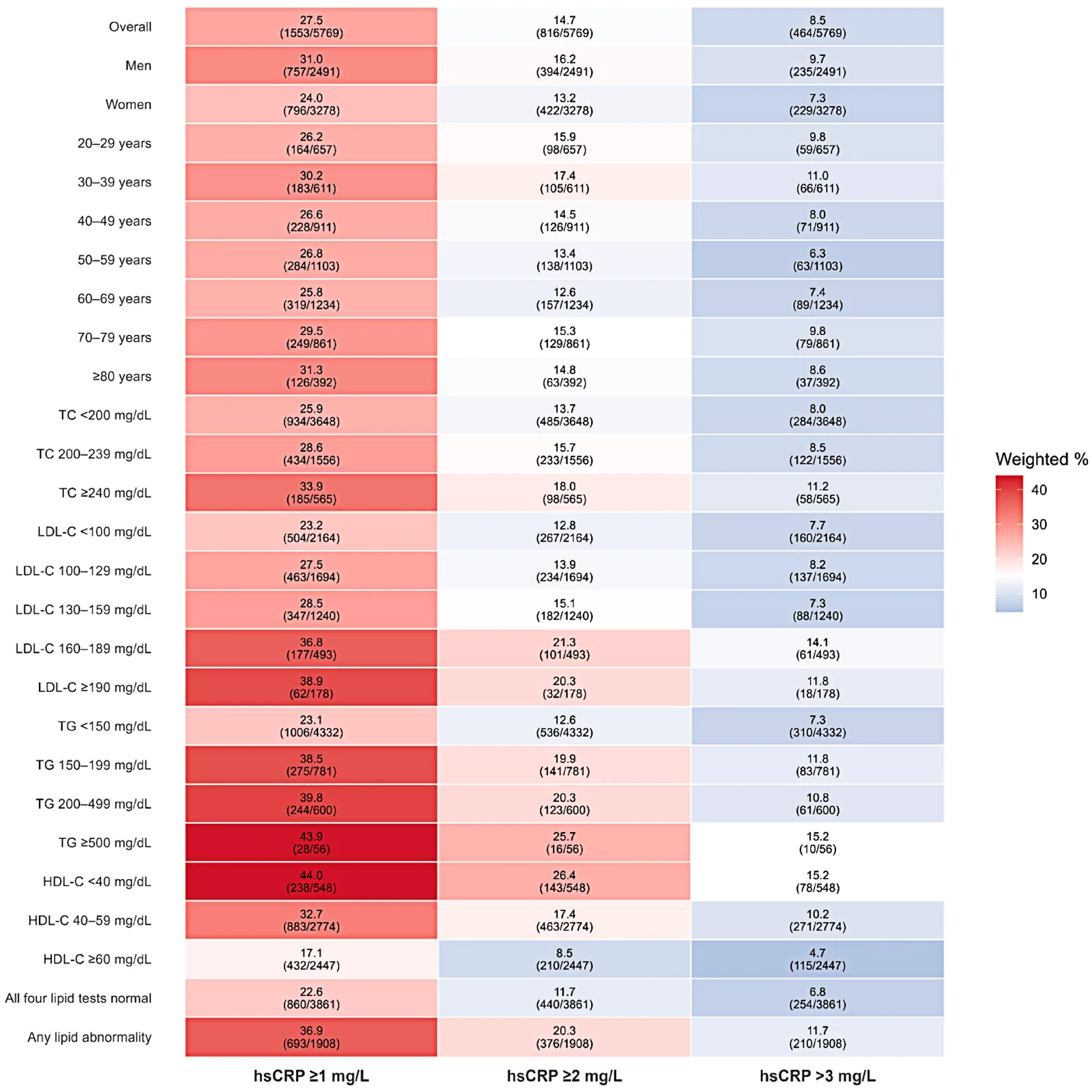
Subgroup-specific prevalence of elevated hsCRP according to demographic and lipid categories. Survey-weighted prevalence of elevated hsCRP was estimated across age, sex, and lipid subgroups. Elevated hsCRP was evaluated using clinically relevant cutoffs, including hsCRP ≥ 1 mg/L, hsCRP ≥ 2 mg/L, and hsCRP > 3 mg/L. Color intensity represents the survey-weighted percentage within each subgroup.

When examined by age group, the weighted prevalence of hsCRP > 3 mg/L varied across 10-year age groups. The survey-weighted prevalence was 9.8% in participants aged 20–29 years, 11.0% in those aged 30–39 years, 8.0% in those aged 40–49 years, 6.3% in those aged 50–59 years, 7.4% in those aged 60–69 years, 9.8% in those aged 70–79 years, and 8.6% in those aged ≥80 years. These findings indicate that elevated hsCRP was not confined to older adults and was also observed among younger adult age groups. The distribution of elevated hsCRP across lipid categories and combined lipid status is shown in the heatmap visualization (Figure 3). The heatmap summarizes the weighted proportions of elevated hsCRP at the ≥1, ≥2, and ≥3 mg/L cutoffs across sex, age group, lipid categories, and combined lipid status.

### 3.2. Additional Detection by hsCRP Beyond Lipid Abnormality

Among the 5769 participants in the primary analysis population, 3861 had normal results for all four lipid tests, corresponding to 66.9% of the unweighted sample and 65.6% of the survey-weighted population. The remaining 1908 participants had at least one lipid abnormality.

In the lipid-normal subgroup, the survey-weighted mean age was 49.7 years, and the survey-weighted proportion of men was 48.4%. The survey-weighted median hsCRP concentration was 0.4 mg/L, with an interquartile range of 0.2–0.9 mg/L. Compared with the overall primary analysis population, the lipid-normal subgroup had a lower survey-weighted mean body mass index and more favorable lipid profiles. The survey-weighted mean body mass index was 23.6 kg/m^2^. The survey-weighted mean concentrations of total cholesterol, LDL-C, triglycerides, and HDL-C were 178.7, 106.4, 96.7, and 62.6 mg/dL, respectively (Table 2).

**Figure 4:**
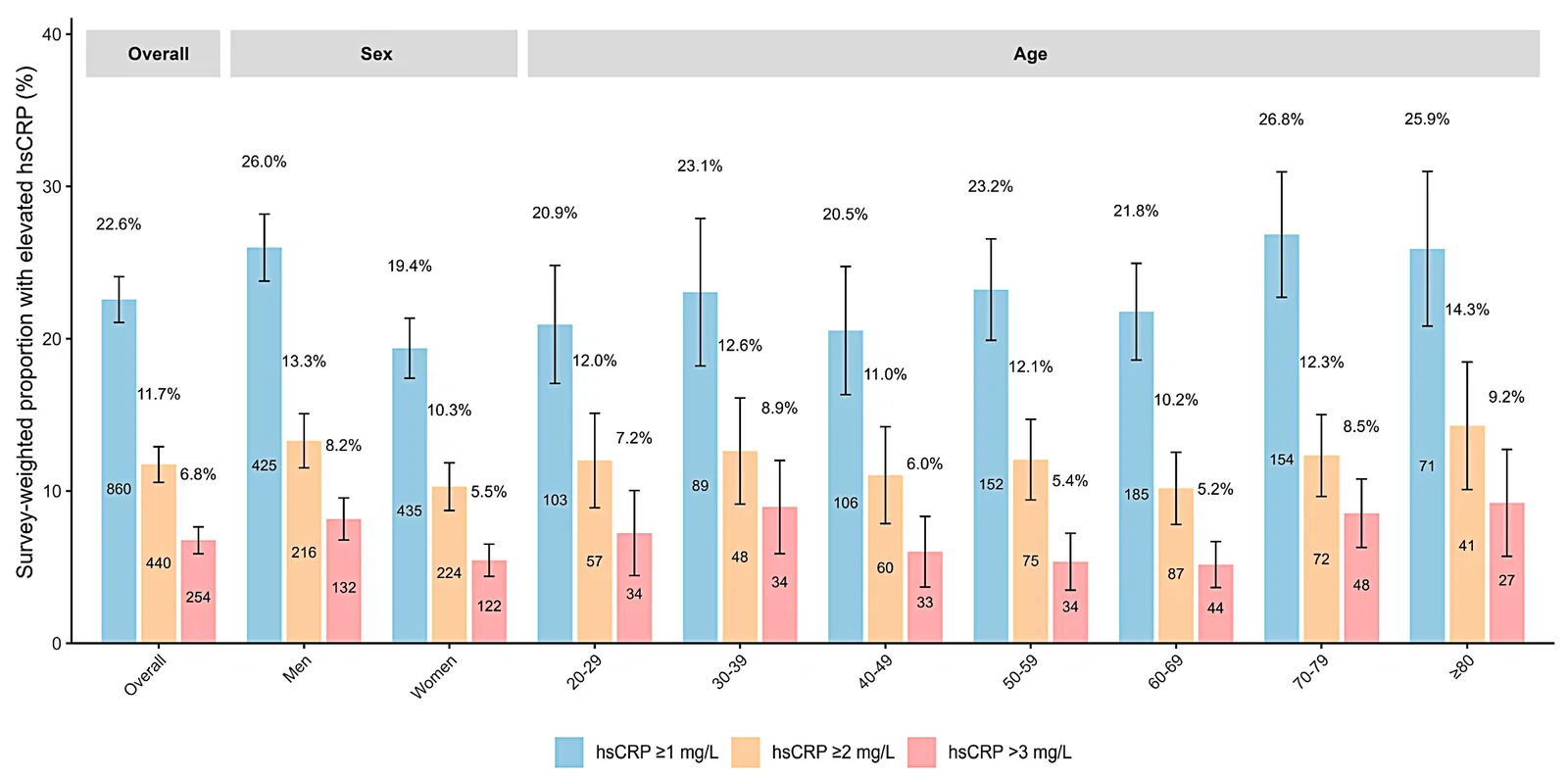
Additional detection of elevated hsCRP at different cutoffs among 3861 lipid-normal Korean adults. Additional detection rates were estimated among participants without conventional lipid abnormalities. Lipid normality was defined as the absence of total cholesterol ≥ 240 mg/dL, directly measured LDL-C ≥ 160 mg/dL, triglycerides ≥ 200 mg/dL, and low HDL-C, defined as HDL-C < 40 mg/dL in men and <50 mg/dL in women. Bars show survey-weighted percentages of lipid-normal participants with hsCRP ≥ 1 mg/L, hsCRP ≥ 2 mg/L, or hsCRP > 3 mg/L. Error bars indicate 95% confidence intervals. Percentages above the bars indicate weighted estimates, while numbers inside the bars indicate the corresponding unweighted numerators. Results are shown for the overall population and according to sex and age group.

Among 3861 participants in this subgroup, the weighted prevalence of elevated hsCRP was 22.6% at the ≥1 mg/L cutoff, 11.7% at the ≥2 mg/L cutoff, and 6.8% at the >3 mg/L cutoff. The weighted prevalence of hsCRP ≥ 10 mg/L in the lipid-normal subgroup was 1.6%, corresponding to 58 participants. Sex-specific patterns were also observed within the lipid-normal subgroup. Among men with all four lipid tests within the normal range, the weighted prevalence of hsCRP ≥1, ≥2, and >3 mg/L was 26.0%, 13.3%, and 8.2%, respectively. Among women with all four lipid tests within the normal range, the corresponding weighted prevalence was 19.4%, 10.3%, and 5.5%, respectively. Age-stratified results in the lipid-normal subgroup showed that hsCRP elevation remained present across all age groups despite normal lipid test results. The weighted prevalence of hsCRP > 3 mg/L was 7.2% in adults aged 20–29 years, 8.9% in those aged 30–39 years, 6.0% in those aged 40–49 years, 5.4% in those aged 50–59 years, 5.2% in those aged 60–69 years, 8.5% in those aged 70–79 years, and 9.2% in those aged ≥80 years. These findings indicate that a measurable proportion of participants with apparently normal lipid profiles had elevated hsCRP (Figure 4).

Among the 5769 participants included in the primary analysis, 3861 had normal results for all four conventional lipid measurements. Within this lipid-normal subgroup, elevated hsCRP was identified in 860, 440, and 254 participants at the ≥1, ≥2, and >3 mg/L cutoffs, respectively. When the entire study population was used as the denominator and the complex survey design was taken into account, the survey-weighted proportions additionally identified by hsCRP testing were 14.8% (95% CI, 13.8–15.9%), 7.7% (95% CI, 6.9–8.5%), and 4.4% (95% CI, 3.8–5.0%), respectively.

### 3.3. Complete Covariate Subgroup and Multivariable Logistic Regression

Subgroup analysis 2 included 5508 participants with complete covariate data. This subgroup was used for survey-weighted multivariable logistic regression analyses of factors associated with elevated hsCRP at the ≥1, ≥2, and >3 mg/L cutoffs. The baseline characteristics of the complete covariate subgroup were generally comparable to those of the primary analysis population, suggesting that the regression subset remained broadly representative of the overall study population (Table 3).

**Figure 5:**
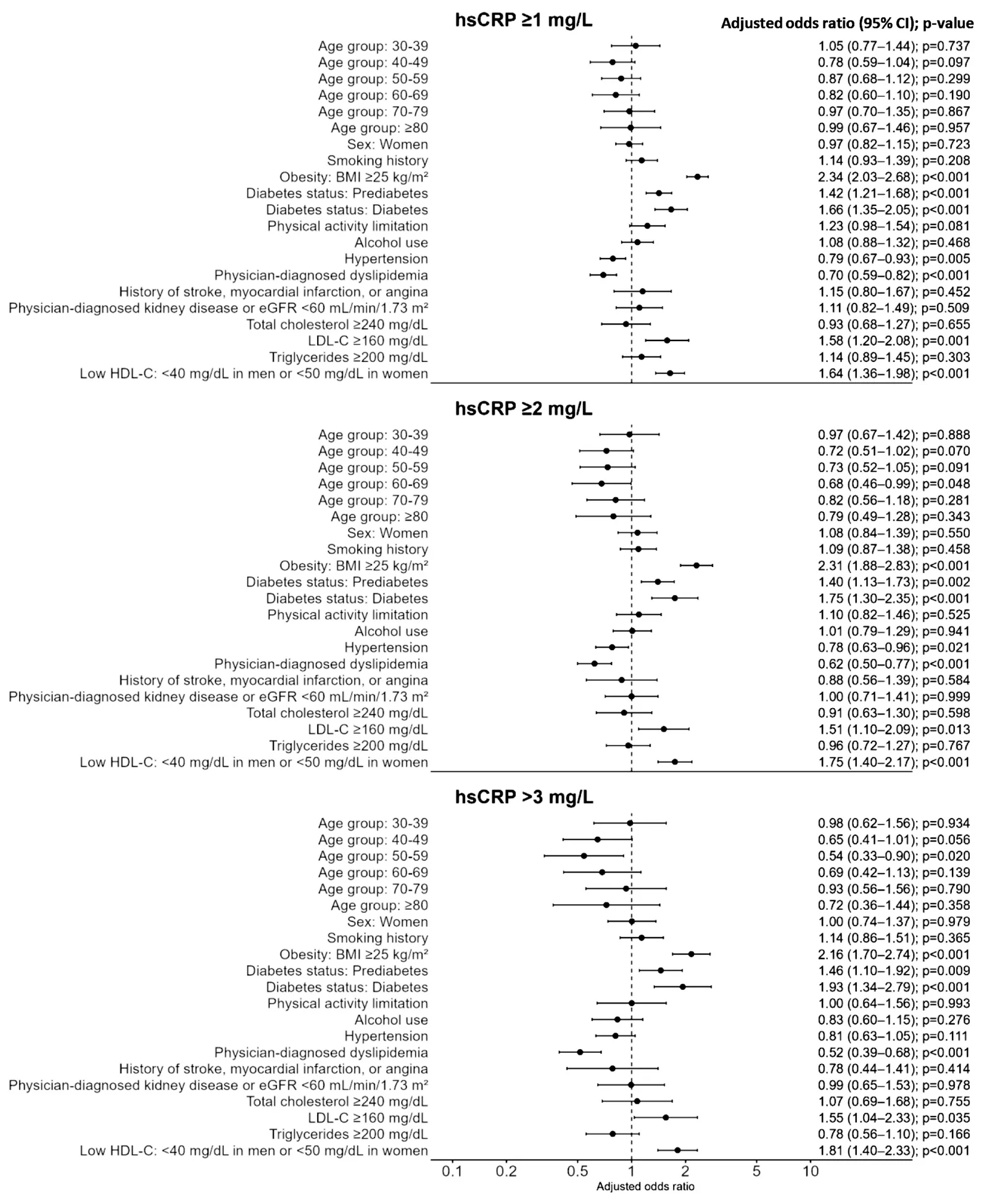
Factors associated with elevated hsCRP levels at different cutoffs: survey-weighted multivariable logistic regression analyses. Survey-weighted adjusted ORs and 95% CIs were estimated using multivariable logistic regression models for hsCRP ≥ 1 mg/L, hsCRP ≥ 2 mg/L, and hsCRP > 3 mg/L. The reference categories were age 20–29 years, men, never-smokers, non-obesity, normal glucose status, no physical activity limitation, no alcohol use, no hypertension, no physician-diagnosed dyslipidemia, no history of cardiovascular disease, no kidney disease, total cholesterol < 240 mg/dL, LDL-C < 160 mg/dL, triglycerides < 200 mg/dL, and HDL-C ≥ 40 mg/dL in men or ≥50 mg/dL in women. Points indicate adjusted odds ratios, and horizontal lines indicate 95% confidence intervals. Abbreviations: BMI, body mass index; CI, confidence interval; eGFR, estimated glomerular filtration rate; HDL-C, high-density lipoprotein cholesterol; hsCRP, high-sensitivity C-reactive protein; LDL-C, low-density lipoprotein cholesterol.

Survey-weighted multivariable logistic regression analyses were performed to evaluate factors independently associated with elevated hsCRP (Figure 5). In survey-weighted multivariable logistic regression analyses, obesity was the factor most consistently associated with elevated hsCRP across all cutoffs, with adjusted odds ratios of 2.34 (95% CI, 2.03–2.68), 2.31 (95% CI, 1.88–2.83), and 2.16 (95% CI, 1.70–2.74) for hsCRP concentrations of ≥1, ≥2, and >3 mg/L, respectively. Diabetes, prediabetes, low HDL-C, and high LDL-C were also independently associated with increased odds of elevated hsCRP at most or all cutoffs. In contrast, physician-diagnosed dyslipidemia was consistently associated with lower odds of elevated hsCRP, while hypertension was inversely associated with hsCRP ≥ 1 and ≥2 mg/L but not with hsCRP > 3 mg/L. The sensitivity analysis excluding participants with hsCRP ≥ 10 mg/L yielded generally similar findings, with obesity, diabetes, low HDL-C, and high LDL-C remaining significantly associated with elevated hsCRP across all cutoffs.

### 3.4. Sensitivity Analysis

Among the 5769 participants included in the primary analysis, 89 participants with hsCRP concentrations ≥ 10 mg/L were excluded, leaving 5680 participants for the sensitivity analysis (Supplementary Table S1). The survey-weighted prevalence of hsCRP concentrations ≥1, ≥2, and >3 mg/L was 26.4% (95% CI, 25.0–27.7%), 13.4% (95% CI, 12.3–14.4%), and 7.0% (95% CI, 6.2–7.9%), respectively. Among the 3803 participants with all four conventional lipid measurements within the normal ranges, the corresponding prevalence estimates were 21.3% (95% CI, 19.9–22.8%), 10.3% (95% CI, 9.2–11.4%), and 5.3% (95% CI, 4.5–6.1%), respectively.

The results of the survey-weighted multivariable logistic regression analyses were generally consistent with those of the primary analysis (Supplementary Figures S1–S3). Obesity, diabetes, high LDL-C, and low HDL-C remained independently associated with elevated hsCRP at all three cutoffs. Prediabetes was associated with hsCRP concentrations ≥1 and ≥2 mg/L but not with hsCRP > 3 mg/L. Physician-diagnosed dyslipidemia was consistently associated with lower odds of elevated hsCRP, whereas hypertension was inversely associated with hsCRP concentrations ≥ 1 and ≥2 mg/L but not with hsCRP > 3 mg/L. Overall, excluding participants with hsCRP concentrations ≥10 mg/L did not materially alter the principal findings.

## 4. Discussion

In this nationally representative analysis of Korean adults using the 2024 KNHANES data, we found that approximately one-quarter of adults had hsCRP ≥ 1 mg/L, 14.7% had hsCRP ≥ 2 mg/L, and 8.5% had hsCRP > 3 mg/L. In contrast, 34.4% of participants had at least one lipid abnormality based on total cholesterol, LDL-C, triglycerides, or sex-specific HDL-C criteria. Importantly, hsCRP identified an additional subset of participants who would not have been classified as having lipid abnormality. Depending on the cutoff, the additional survey-weighted detection by hsCRP ranged from 4.4% to 14.8%. These findings suggest that hsCRP may capture an inflammatory risk phenotype that is not fully reflected by conventional lipid measurements [1,2,3,4].

Compared with Western general populations, Korean adults in the present study showed a substantially lower prevalence of elevated hsCRP across all commonly used cutoffs. In the UK Biobank, 59.2% of adults without known ASCVD had hsCRP ≥ 1 mg/L, 33.6% had hsCRP ≥ 2 mg/L, and 21.2% had hsCRP > 3 mg/L. In contrast, the corresponding proportions in the present Korean population were 27.5%, 14.7%, and 8.5%, respectively [7]. Prior US NHANES-based analyses have also suggested that approximately half of US adults have hsCRP ≥ 2 mg/L [1,24,25]. These differences support the concept that hsCRP distributions are shifted downward in East Asian populations, although elevated hsCRP may still provide prognostically relevant information within this lower range [1,2,3,4,5]. The observed distribution of hsCRP in Korean adults appears lower than that reported in several Western populations [7,10,11]. In previous analyses of US adults, CRP concentrations were reported to vary substantially by race and ethnicity [1,24]. Compared with these reports, the proportion of Korean adults with hsCRP > 3 mg/L in the present study was relatively modest. This is consistent with previous multiethnic observations suggesting that East Asian populations tend to have lower CRP concentrations than Black, Hispanic, South Asian, or White populations [1,2,3,4,7,10,11].

Several potential mechanisms may explain these ethnic and population differences. First, differences in obesity prevalence and body composition are likely important, because adiposity is a major determinant of low-grade systemic inflammation [1,2,3,4,5,26,27]. Previous multiethnic studies have shown that adjustment for body mass index and other metabolic factors attenuates, but does not completely eliminate, racial and ethnic differences in CRP [26,27]. Second, genetic background, lifestyle factors, dietary patterns, socioeconomic factors, smoking exposure, and the prevalence of metabolic syndrome may contribute to population-level differences in hsCRP distribution [1,2,3,4,7,28]. Third, differences in study design, assay methods, exclusion criteria, and the handling of participants with CRP ≥ 10 mg/L may influence the reported distribution [29].

The incremental detection analysis is clinically relevant because lipid abnormalities and systemic inflammation represent related but non-identical domains of cardiovascular risk [1,2,3,4,7]. The largest incremental detection was observed using hsCRP ≥ 1 mg/L, which added 14.8% beyond lipid abnormality. This threshold may be useful for describing low-grade inflammatory burden at the population level, although its clinical actionability is less direct than that of hsCRP ≥ 2 mg/L or >3 mg/L [1,2,3,4,7,9]. Conversely, hsCRP > 3 mg/L identified a smaller but potentially higher-risk group, adding 4.4% beyond lipid abnormality. These findings indicate that the choice of hsCRP cutoff should depend on the intended purpose: hsCRP ≥ 1 mg/L may be more sensitive for population surveillance, hsCRP ≥ 2 mg/L may align better with cardiovascular risk-enhancing frameworks, and hsCRP > 3 mg/L may identify a smaller group with more pronounced inflammatory burden [1,2,3,4,7,9,11].

The strong and consistent association between lower HDL-C and elevated hsCRP is consistent with previous Korean population-based data showing that low HDL-C is more prevalent among individuals with high hsCRP [30,31,32,33]. This relationship may be explained by shared metabolic-inflammatory pathways, because low HDL-C commonly accompanies insulin resistance, adiposity, hypertriglyceridemia, and low-grade inflammation [30,31,32,33]. The association observed in our study may reflect both low HDL-C as a marker of adverse cardiometabolic status and inflammation-related impairment of HDL function [33]. The findings may support the concept that hsCRP elevation is closely linked to the low-HDL phenotype within the broader cardiometabolic risk profile [1,2,3,4,7,30,31,32,33]. Beyond conventional lipid measurements, residual cardiovascular risk may reflect both persistent inflammation and unmeasured atherogenic lipoprotein burden [1,2,3,4,5,6,7]. hsCRP provides information on residual inflammatory risk, whereas apolipoprotein B, non-HDL cholesterol, lipoprotein(a), and remnant cholesterol may capture complementary aspects of lipid-related residual risk that are not fully reflected by LDL-C alone [24,25]. Future studies incorporating these biomarkers together with hsCRP and prospective cardiovascular outcomes may enable a more comprehensive assessment of cardiovascular risk. Prospective Korean cohort studies are needed to determine whether hsCRP independently predicts incident cardiovascular events and provides incremental prognostic value beyond conventional lipid measurements [7].

This study has several strengths. First, we used a nationally representative dataset and applied the complex survey design, including sampling weights, stratification, and primary sampling units [16,17]. Second, hsCRP was evaluated using both mutually exclusive categories and clinically relevant cutoffs, allowing both distributional and risk-oriented interpretation. Third, lipid abnormality was defined using four commonly used lipid criteria, and the incremental contribution of hsCRP was quantified beyond this composite lipid abnormality definition [22,23]. Given the lower hsCRP concentrations reported in Korean populations and the lack of specified hsCRP cutoffs in Korean dyslipidemia guidelines, this study provides updated nationally representative data on hsCRP distribution and the additional proportion identified at different thresholds among adults without lipid abnormalities.

Several limitations should also be acknowledged. First, because this was a cross-sectional analysis, causal relationships between lipid abnormalities, hsCRP, and cardiovascular outcomes (e.g., myocardial infarction, stroke, cardiovascular mortality, or major adverse cardiovascular events) could not be assessed. Therefore, our findings should be interpreted as demonstrating additional identification of individuals with elevated hsCRP rather than improved cardiovascular risk prediction. The clinical implications of incremental hsCRP detection require validation in longitudinal Korean cohorts [4,10,14]. Second, hsCRP is a nonspecific marker of inflammation and may be influenced by acute infection, chronic inflammatory disease, medication use, smoking, obesity, and other unmeasured factors [1,2,3,4,5,6,7]. Although several relevant factors available in the publicly accessible KNHANES database were included as covariates, detailed clinical histories and information on other potential confounders could not be assessed. Because hsCRP was evaluated using predefined cutoffs, its association with relevant factors across the full continuous range and potential effect modification by age or sex could not be assessed. These issues warrant further investigation. Finally, the definition of lipid abnormality was based on selected lipid cutoffs, and different definitions, such as non-HDL-C, apolipoprotein B, or guideline-based risk categories, could yield different estimates of incremental detection [1,2,3,4,7,12].

## 5. Conclusions

In conclusion, this nationally representative analysis showed that elevated hsCRP is present in a meaningful proportion of Korean adults and identifies additional individuals who are not detected by conventional lipid abnormalities. The relatively modest prevalence of hsCRP > 3 mg/L compared with Western populations is consistent with prior evidence suggesting lower CRP concentrations in East Asian populations. Nevertheless, the incremental detection by hsCRP supports its potential role as a complementary inflammatory biomarker in cardiovascular risk assessment among Korean adults. However, given the cross-sectional design and lack of cardiovascular outcome data, our findings indicate additional identification of elevated hsCRP rather than improved cardiovascular risk classification. Future longitudinal studies are needed to determine whether hsCRP improves risk prediction and clinical decision-making beyond lipid measurements in the Korean population.

## Figures and Tables

**Table 1:** Baseline characteristics of 5769 KNHANES subjects.

Characteristics	Results
Age, years	50.0 ± 0.5
Body mass index, kg/m^2^	24.3 ± 0.1
Total cholesterol, mg/dL	189.3 ± 0.7
LDL-C, mg/dL	116.7 ± 0.7
Triglycerides, mg/dL	131.1 ± 1.7
HDL-C, mg/dL	57.8 ± 0.3
eGFR, mL/min/1.73 m^2^	95.9 ± 0.4
hsCRP, mg/L	0.50 (0.30–1.10)
Sex	
Men	2491/5769 (50.4; 49.2–51.5)
Women	3278/5769 (49.6; 48.5–50.8)
Age group	
20–29 years	657/5769 (14.6; 13.0–16.2)
30–39 years	611/5769 (15.5; 13.9–17.1)
40–49 years	911/5769 (18.0; 16.4–19.7)
50–59 years	1103/5769 (20.1; 18.8–21.5)
60–69 years	1234/5769 (17.5; 15.9–19.0)
70–79 years	861/5769 (9.6; 8.5–10.7)
≥80 years	392/5769 (4.6; 3.9–5.4)
Smoking status: Smoker ^1^	2112/5768 (40.6; 38.9–42.2)
Obesity: BMI ≥ 25 kg/m^2^	2082/5680 (37.7; 36.2–39.2)
Diabetes status: Prediabetes	1841/5587 (29.8; 28.2–31.4)
Diabetes status: Diabetes	958/5587 (14.4; 13.2–15.6)
Physical activity limitation: Limited	667/5768 (9.5; 8.3–10.6)
Alcohol use	4567/5768 (81.4; 80.1–82.7)
Hypertension	2077/5768 (30.9; 29.1–32.7)
Physician-diagnosed dyslipidemia	1631/5768 (24.0; 22.5–25.5)
Cardiovascular disease history (stroke, myocardial infarction, or angina)	277/5303 (3.6; 3.1–4.1)
Physician-diagnosed kidney disease or eGFR < 60 mL/min/1.73 m^2^	357/5768 (4.9; 4.3–5.5)
High total cholesterol: Total cholesterol ≥ 240 mg/dL	565/5769 (10.6; 9.6–11.6)
High LDL-C: LDL-C ≥ 160 mg/dL	671/5769 (12.8; 11.6–14.0)
High triglycerides: Triglycerides ≥ 200 mg/dL	656/5769 (12.8; 11.8–13.9)
Low HDL-C: HDL-C < 40 mg/dL in men or <50 mg/dL in women	1058/5769 (17.8; 16.5–19.2)
All four lipid tests normal	3861/5769 (65.6; 64.2–67.0)
Any lipid abnormality	1908/5769 (34.4; 33.0–35.8)
hsCRP ≥10 mg/L	89/5769 (1.6; 1.2–1.9)

^1^ Smoking history was defined as any smoking history other than never-smoking. Continuous variables are presented as survey-weighted mean ± standard error, except for hsCRP, which is presented as survey-weighted median and interquartile range. Categorical variables are presented as unweighted n/N and survey-weighted percentage with 95% confidence interval. Denominators differ across variables because some covariate information was missing. The primary analysis population included participants with hsCRP concentrations ≥ 10 mg/L. eGFR, estimated glomerular filtration rate; HDL-C, high-density lipoprotein cholesterol; hsCRP, high-sensitivity C-reactive protein; LDL-C, low-density lipoprotein cholesterol.

**Table 2:** Characteristics of 3861 participants with normal results for all four lipid tests.

Characteristics	Results
Age, years	49.7 ± 0.5
Body mass index, kg/m^2^	23.6 ± 0.1
Total cholesterol, mg/dL	178.7 ± 0.5
LDL-C, mg/dL	106.4 ± 0.5
Triglycerides, mg/dL	96.7 ± 0.8
HDL-C, mg/dL	62.6 ± 0.3
eGFR, mL/min/1.73 m^2^	96.7 ± 0.5
hsCRP, mg/L	0.40 (0.20–0.90)
Sex	
Men	1636/3861 (48.4; 46.9–49.9)
Women	2225/3861 (51.6; 50.1–53.1)
Age group	
20–29 years	528/3861 (17.4; 15.4–19.4)
30–39 years	382/3861 (14.1; 12.4–15.9)
40–49 years	570/3861 (16.7; 14.9–18.6)
50–59 years	684/3861 (18.7; 17.2–20.3)
60–69 years	836/3861 (18.4; 16.6–20.1)
70–79 years	600/3861 (10.0; 8.8–11.2)
≥80 years	261/3861 (4.6; 3.8–5.4)
Smoking status: Smoker ^1^	1364/3757 (38.5; 36.7–40.3)
Obesity: BMI ≥ 25 kg/m^2^	1151/3813 (30.3; 28.5–32.1)
Diabetes status: Prediabetes	1164/3861 (27.6; 25.9–29.4)
Diabetes status: Diabetes	611/3861 (13.5; 12.2–14.8)
Physical activity limitation: Limited	440/3860 (9.2; 8.0–10.5)
Alcohol use	3083/3861 (82.3; 80.8–83.8)
Hypertension	3031/3861 (77.8; 76.5–79.1)
Physician-diagnosed dyslipidemia	1113/3860 (24.6; 22.8–26.3)
Cardiovascular disease history (stroke, myocardial infarction, or angina)	192/3860 (3.7; 3.2–4.3)
Physician-diagnosed kidney disease or eGFR < 60 mL/min/1.73 m^2^	215/3861 (4.4; 3.7–5.1)
High total cholesterol: Total cholesterol ≥ 240 mg/dL	0/3861 (0.0; 0.0–0.0)
High LDL-C: LDL-C ≥ 160 mg/dL	0/3861 (0.0; 0.0–0.0)
High triglycerides: Triglycerides ≥ 200 mg/dL	0/3861 (0.0; 0.0–0.0)
Low HDL-C: HDL-C < 40 mg/dL in men or <50 mg/dL in women	0/3861 (0.0; 0.0–0.0)
All four lipid tests normal	3861/3861 (100.0; 100.0–100.0)
Any lipid abnormality	0/3861 (0.0; 0.0–0.0)
hsCRP ≥ 10 mg/L	58/3861 (1.6; 1.1–2.0)

^1^ Smoking history was defined as any smoking history other than never-smoking. Continuous variables are presented as survey-weighted mean ± standard error, except for hsCRP, which is presented as survey-weighted median and interquartile range. Categorical variables are presented as unweighted n/N and survey-weighted percentage with 95% confidence interval. Denominators differ across variables because some covariate information was missing. The primary analysis population included participants with hsCRP concentrations ≥ 10 mg/L. BMI, body mass index; eGFR, estimated glomerular filtration rate; HDL-C, high-density lipoprotein cholesterol; hsCRP, high-sensitivity C-reactive protein; LDL-C, low-density lipoprotein cholesterol.

**Table 3:** Characteristics of 5508 participants with complete covariate information.

Characteristics	Results
Age, years	49.6 ± 0.5
Body mass index, kg/m^2^	24.3 ± 0.1
Total cholesterol, mg/dL	189.5 ± 0.7
LDL-C, mg/dL	116.9 ± 0.7
Triglycerides, mg/dL	130.9 ± 1.8
HDL-C, mg/dL	57.9 ± 0.3
eGFR, mL/min/1.73 m^2^	96.2 ± 0.4
hsCRP, mg/L	0.50 (0.30–1.00)
Sex	
Men	2383/5508 (50.5; 49.4–51.7)
Women	3125/5508 (49.5; 48.3–50.6)
Age group	
20–29 years	642/5508 (14.9; 13.2–16.5)
30–39 years	599/5508 (15.8; 14.2–17.5)
40–49 years	877/5508 (18.0; 16.3–19.7)
50–59 years	1065/5508 (20.3; 18.9–21.7)
60–69 years	1202/5508 (17.7; 16.1–19.3)
70–79 years	814/5508 (9.5; 8.4–10.5)
≥80 years	309/5508 (3.8; 3.2–4.4)
Smoking status: Smoker ^1^	2067/5508 (41.4; 39.7–43.1)
Obesity: BMI ≥ 25 kg/m^2^	2025/5508 (38.2; 36.7–39.7)
Diabetes status: Prediabetes	1822/5508 (30.7; 29.1–32.3)
Diabetes status: Diabetes	926/5508 (14.7; 13.5–15.9)
Physical activity limitation: Limited	638/5508 (9.4; 8.3–10.6)
Alcohol use	4497/5508 (83.5; 82.3–84.7)
Hypertension	4236/5508 (75.7; 74.6–76.8)
Physician-diagnosed dyslipidemia	1547/5508 (23.8; 22.3–25.3)
Cardiovascular disease history (stroke, myocardial infarction, or angina)	259/5508 (3.6; 3.1–4.1)
Physician-diagnosed kidney disease or eGFR < 60 mL/min/1.73 m^2^	325/5508 (4.7; 4.1–5.3)
High total cholesterol: Total cholesterol ≥ 240 mg/dL	540/5508 (10.6; 9.6–11.6)
High LDL-C: LDL-C ≥ 160 mg/dL	644/5508 (12.8; 11.6–14.0)
High triglycerides: Triglycerides ≥ 200 mg/dL	625/5508 (12.9; 11.8–14.0)
Low HDL-C: HDL-C < 40 mg/dL in men or <50 mg/dL in women	997/5508 (17.6; 16.2–19.0)
All four lipid tests normal	3702/5508 (65.9; 64.4–67.3)
Any lipid abnormality	1806/5508 (34.1; 32.7–35.6)
hsCRP ≥ 10 mg/L	85/5508 (1.5; 1.2–1.9)

^1^ Smoking history was defined as any smoking history other than never-smoking. Continuous variables are presented as survey-weighted mean ± standard error, except for hsCRP, which is presented as survey-weighted median and interquartile range. Categorical variables are presented as unweighted n/N and survey-weighted percentage with 95% confidence interval. Denominators differ across variables because some covariate information was missing. The primary analysis population included participants with hsCRP concentrations ≥ 10 mg/L. BMI, body mass index; eGFR, estimated glomerular filtration rate; HDL-C, high-density lipoprotein cholesterol; hsCRP, high-sensitivity C-reactive protein; LDL-C, low-density lipoprotein cholesterol.

## Data Availability

The datasets generated and analyzed during the current study are available from the public database of KNHANES https://knhanes.kdca.go.kr/knhanes/eng/main.do, accessed on 16 July 2026.

## Supplementary Materials

The following supporting information can be downloaded at: https://www.mdpi.com/article/10.3390/metabo16080531/s1, Figure S1: Subgroup-specific prevalence of elevated hsCRP according to demographic and lipid categories in the sensitivity analysis population. Survey-weighted prevalence of elevated hsCRP was estimated across age, sex, and lipid subgroups. Elevated hsCRP was evaluated using clinically relevant cut-offs, including hsCRP ≥ 1 mg/L, hsCRP ≥2 mg/L, and hsCRP > 3 mg/L. Color intensity represents the survey-weighted percentage within each subgroup; Figure S2: Additional detection of elevated hsCRP at different cutoffs among lipid-normal participants in the sensitivity analysis population. Additional detection rates were estimated among participants without conventional lipid abnormalities. Lipid normality was defined as the absence of total cholesterol ≥240 mg/dL, directly measured LDL-C ≥ 160 mg/dL, triglycerides ≥ 200 mg/dL, and low HDL-C, defined as HDL-C < 40 mg/dL in men and <50 mg/dL in women. Bars show survey-weighted percentages of lipid-normal participants with hsCRP ≥ 1 mg/L, hsCRP ≥ 2 mg/L, or hsCRP > 3 mg/L. Error bars indicate 95% confidence intervals. Percentages above the bars indicate weighted estimates, while numbers inside the bars indicate the corresponding unweighted numerators. Results are shown for the overall population and according to sex and age group; Figure S3: Factors associated with elevated hsCRP levels at different cutoffs in the sensitivity analysis population: survey-weighted multivariable logistic regression analyses. Survey-weighted adjusted ORs and 95% CIs were estimated using multivariable logistic regression models for hsCRP ≥ 1 mg/L, hsCRP ≥ 2 mg/L, and hsCRP > 3 mg/L. The reference categories were age 20–29 years, men, never-smokers, non-obesity, normal glucose status, no physical activity limitation, no alcohol use, no hypertension, no physician-diagnosed dyslipidemia, no history of cardiovascular disease, no kidney disease, total cholesterol < 240 mg/dL, LDL-C < 160 mg/dL, triglycerides < 200 mg/dL, and HDL-C ≥40 mg/dL in men or ≥50 mg/dL in women. Points indicate adjusted odds ratios, and horizontal lines indicate 95% confidence intervals. Abbreviations: BMI, body mass index; CI, confidence interval; eGFR, estimated glomerular filtration rate; HDL-C, high-density lipoprotein cholesterol; hsCRP, high-sensitivity C-reactive protein; LDL-C, low-density lipoprotein cholesterol; Table S1: Characteristics of the sensitivity analysis population after excluding participants with hsCRP concentrations ≥ 10 mg/L. ^1^ Smoking history was defined as any smoking history other than never-smoking. Continuous variables are presented as survey-weighted mean ± standard error, except for hsCRP, which is presented as survey-weighted median and interquartile range. Categorical variables are presented as un-weighted n/N and survey-weighted percentage with 95% confidence interval. Denominators differ across variables because some covariate information was missing. The primary analysis population included participants with hsCRP concentrations ≥ 10 mg/L. BMI, body mass index; eGFR, estimated glomerular filtration rate; HDL-C, high-density lipoprotein cholesterol; hsCRP, high-sensitivity C-reactive protein; LDL-C, low-density lipoprotein cholesterol.

## Abbreviations

The following abbreviations are used in this manuscript:

AHA	American Hospital Association
CI	Confidence Interval
HDL-C	High-density lipoprotein cholesterol
hsCRP	High-sensitivity C-reactive protein
KNHANES	Korea National Health and Nutrition Examination Survey
LDL-C	Low-density lipoprotein cholesterol
US CDC	United States of America Centers for Disease Control and Prevention
