# Supplementary material for: Impact of High-Sensitivity C-Reactive Protein Cutoff Selection on Cardiovascular Risk Classification Beyond Lipid Measurements in Korean Adults: A KNHANES 2024 Study
Source: Metabolites. 2026 Jul 27;16(8):531. doi: 10.3390/metabo16080531 (PMC13515918; doi:10.3390/metabo16080531)
Supplement: Supplementary file 1 [file metabolites-16-00531-s001.zip › metabolites-4415932-supplementary.pdf]

metabolites

MDPI

Table S1. Characteristics of the sensitivity analysis population after excluding participants with hsCRP concentrations ≥10 mg/L.

**Characteristics**	**Sensitivity analysis population all**	**Sensitivity subgroup analysis 1: all four lipid tests normal**	**Sensitivity subgroup analysis 2: complete covariate information**
Age, years	50.0 ± 0.5	49.7 ± 0.5	49.6 ± 0.5
Body mass index, kg/m²	24.3 ± 0.1	23.6 ± 0.1	24.3 ± 0.1
Total cholesterol, mg/dL	189.5 ± 0.7	178.9 ± 0.5	189.7 ± 0.7
LDL-C, mg/dL	116.8 ± 0.7	106.5 ± 0.5	117.0 ± 0.7
Triglycerides, mg/dL	131.4 ± 1.8	96.8 ± 0.8	131.1 ± 1.8
HDL-C, mg/dL	57.9 ± 0.3	62.6 ± 0.3	58.0 ± 0.3
eGFR, mL/min/1.73 m²	95.9 ± 0.4	96.7 ± 0.4	96.2 ± 0.4
hsCRP, mg/L	0.50 (0.30–1.00)	0.40 (0.20–0.80)	0.50 (0.20–1.00)
Sex			
Men	2,445/5,680 (50.3; 49.1–51.4)	1,603/3,803 (48.2; 46.7–49.7)	2,339/5,423 (50.4; 49.3–51.6)
Women	3,235/5,680 (49.7; 48.6–50.9)	2,200/3,803 (51.8; 50.3–53.3)	3,084/5,423 (49.6; 48.4–50.7)
Age group			
20-29 years	644/5,680 (14.5; 12.9–16.1)	517/3,803 (17.3; 15.3–19.3)	630/5,423 (14.8; 13.2–16.4)
30-39 years	603/5,680 (15.6; 13.9–17.2)	374/3,803 (14.1; 12.3–15.9)	591/5,423 (15.9; 14.2–17.6)
40-49 years	896/5,680 (18.1; 16.4–19.7)	563/3,803 (16.8; 15.0–18.6)	862/5,423 (18.0; 16.3–19.7)
50-59 years	1,096/5,680 (20.3; 18.9–21.7)	680/3,803 (18.9; 17.4–20.4)	1,058/5,423 (20.5; 19.1–21.9)
60-69 years	1,216/5,680 (17.5; 15.9–19.0)	827/3,803 (18.4; 16.7–20.2)	1,185/5,423 (17.7; 16.1–19.3)
70-79 years	839/5,680 (9.5; 8.4–10.6)	584/3,803 (9.9; 8.7–11.1)	793/5,423 (9.4; 8.3–10.4)
≥80 years	386/5,680 (4.6; 3.9–5.3)	258/3,803 (4.6; 3.8–5.4)	304/5,423 (3.8; 3.2–4.4)
Smoking status: Smoker[1]	2,075/5,520 (40.5; 38.9–42.2)	1,345/3,701 (38.6; 36.8–40.5)	2,035/5,423 (41.5; 39.8–43.1)
Obesity: BMI ≥25 kg/m²	2,047/5,593 (37.6; 36.1–39.2)	1,130/3,756 (30.2; 28.4–31.9)	1,990/5,423 (38.1; 36.6–39.7)
Diabetes status: Prediabetes	1,870/5,680 (30.7; 29.1–32.2)	1,141/3,803 (27.5; 25.8–29.2)	1,787/5,423 (30.6; 29.0–32.2)
Diabetes status: Diabetes	978/5,680 (14.9; 13.7–16.1)	601/3,803 (13.5; 12.2–14.8)	906/5,423 (14.5; 13.3–15.8)
Physical activity limitation: Limited	655/5,679 (9.4; 8.3–10.6)	431/3802 (9.2; 8.0–10.4)	628/5,423 (9.5; 8.3–10.6)
Alcohol use	4,501/5,680 (81.5; 80.1–82.8)	3,038/3,803 (82.2; 80.7–83.7)	4,433/5,423 (83.6; 82.4–84.8)
Hypertension	4,350/5,642 (75.3; 74.2–76.4)	2,984/3,780 (77.8; 76.4–79.1)	4,169/5,423 (75.7; 74.6–76.8)
Physician-diagnosed dyslipidemia	1,606/5,679 (24.0; 22.5–25.5)	1,097/3,802 (24.6; 22.9–26.3)	1,525/5,423 (23.8; 22.3–25.3)

 https://doi.org/10.3390/metabo16080531

Cardiovascular disease history (stroke, myocardial infarction, or angina)	273/5,679 (3.6; 3.1–4.1)	189/3,802 (3.7; 3.1–4.3)	255/5,423 (3.6; 3.1–4.1)
Physician-diagnosed kidney disease or eGFR <60 mL/min/1.73 m^2	349/5,680 (4.8; 4.2–5.4)	209/3,803 (4.3; 3.6–5.1)	317/5,423 (4.6; 4.0–5.2)
High total cholesterol: Total cholesterol ≥240 mg/dL	558/5,680 (10.6; 9.6–11.6)	0/3,803 (0.0; 0.0–0.0)	533/5,423 (10.7; 9.6–11.7)
High LDL-C: LDL-C ≥160 mg/dL	662/5,680 (12.8; 11.7–14.0)	0/3,803 (0.0; 0.0–0.0)	635/5,423 (12.8; 11.6–14.0)
High triglycerides: Triglycerides ≥200 mg/dL	652/5,680 (13.0; 11.9–14.0)	0/3,803 (0.0; 0.0–0.0)	621/5,423 (13.0; 11.8–14.1)
Low HDL-C: HDL-C <40 mg/dL in men or <50 mg/dL in women	1,032/5,680 (17.7; 16.3–19.0)	0/3,803 (0.0; 0.0–0.0)	972/5,423 (17.5; 16.0–18.9)
All four lipid tests normal	3,803/5,680 (65.6; 64.2–67.0)	3,803/3,803 (100.0; 100.0–100.0)	3,647/5,423 (65.9; 64.4–67.3)
Any lipid abnormality	1,877/5,680 (34.4; 33.0–35.8)	0/3,803 (0.0; 0.0–0.0)	1,776/5,423 (34.1; 32.7–35.6)
hsCRP ≥10 mg/L	0/5,680 (0.0; 0.0–0.0)	0/3,803 (0.0; 0.0–0.0)	0/5,423 (0.0; 0.0–0.0)

[1]Smoking history was defined as any smoking history other than never-smoking. Continuous variables are presented as survey-weighted mean ± standard error, except for hsCRP, which is presented as survey-weighted median and interquartile range. Categorical variables are presented as unweighted n/N and survey-weighted percentage with 95% confidence interval. Denominators differ across variables because some covariate information was missing. The primary analysis population included participants with hsCRP concentrations ≥10 mg/L. BMI, body mass index; eGFR, estimated glomerular filtration rate; HDL-C, high-density lipoprotein cholesterol; hsCRP, high-sensitivity C-reactive protein; LDL-C, low-density lipoprotein cholesterol.

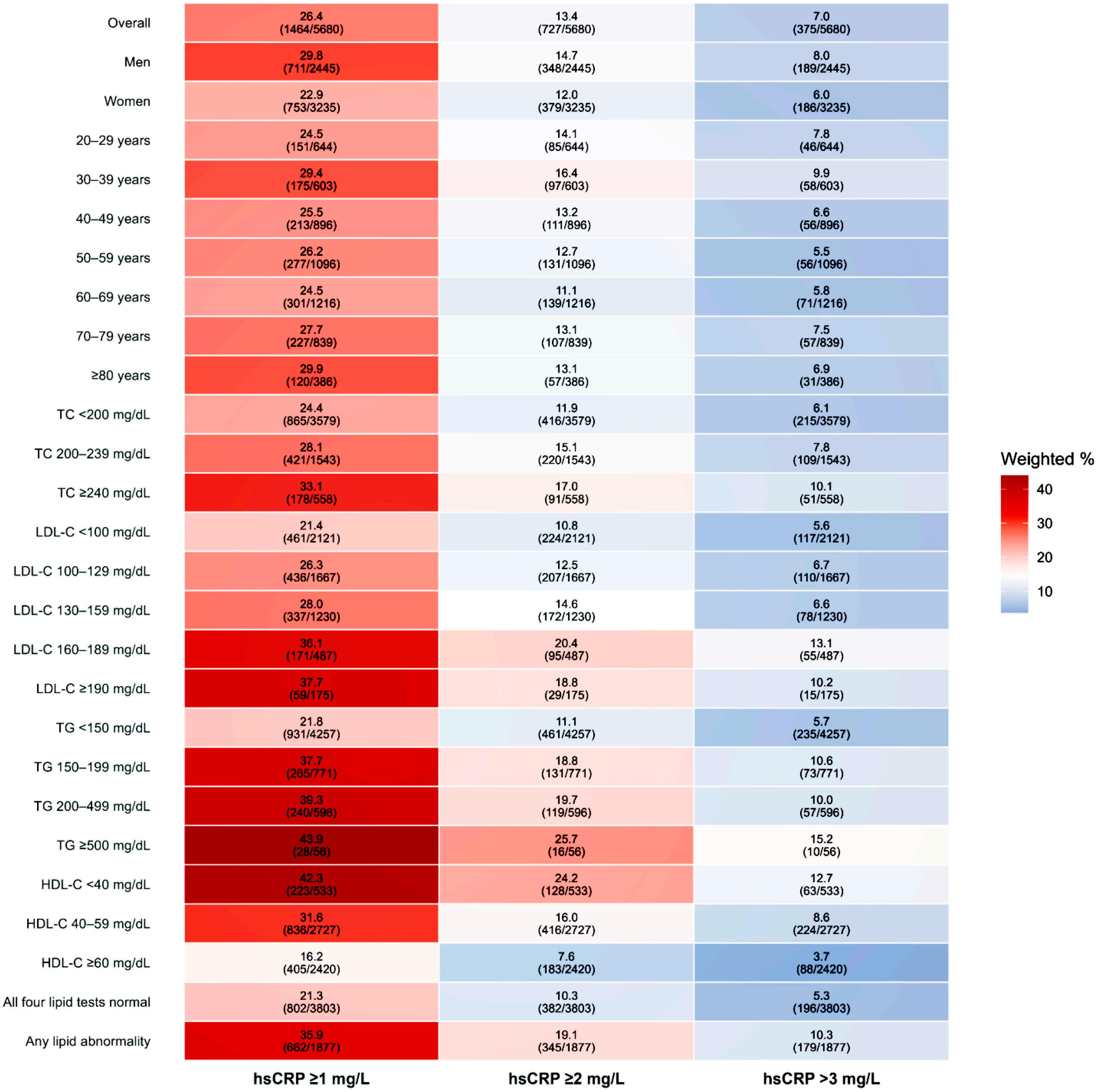

Figure S1. Subgroup-specific prevalence of elevated hsCRP according to demographic and lipid categories in the sensitivity analysis population. Survey-weighted prevalence of elevated hsCRP was estimated across age, sex, and lipid subgroups. Elevated hsCRP was evaluated using clinically relevant cutoffs, including hsCRP ≥1 mg/L, hsCRP ≥2 mg/L, and hsCRP >3 mg/L. Color intensity represents the survey-weighted percentage within each subgroup.

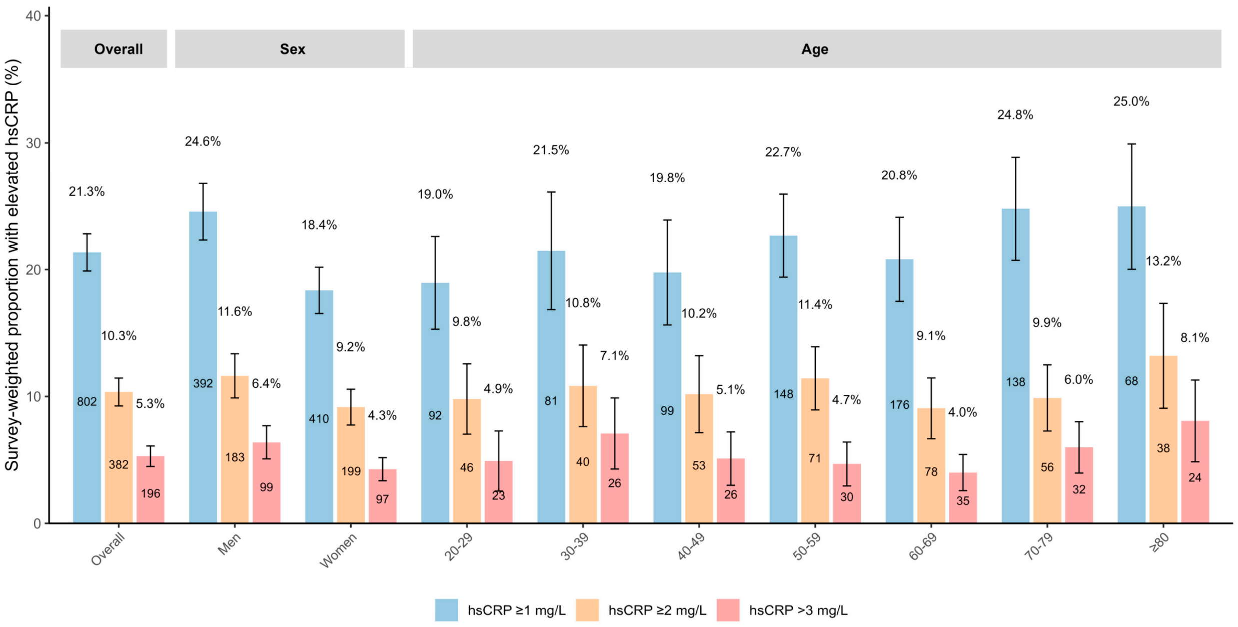

Figure S2. Additional detection of elevated hsCRP at different cutoffs among lipid-normal participants in the sensitivity analysis population. Additional detection rates were estimated among participants without conventional lipid abnormalities. Lipid normality was defined as the absence of total cholesterol ≥240 mg/dL, directly measured LDL-C ≥160 mg/dL, triglycerides ≥200 mg/dL, and low HDL-C, defined as HDL-C <40 mg/dL in men and <50 mg/dL in women. Bars show survey-weighted percentages of lipid-normal participants with hsCRP ≥1 mg/L, hsCRP ≥2 mg/L, or hsCRP >3 mg/L. Error bars indicate 95% confidence intervals. Percentages above the bars indicate weighted estimates, while numbers inside the bars indicate the corresponding unweighted numerators. Results are shown for the overall population and according to sex and age group.

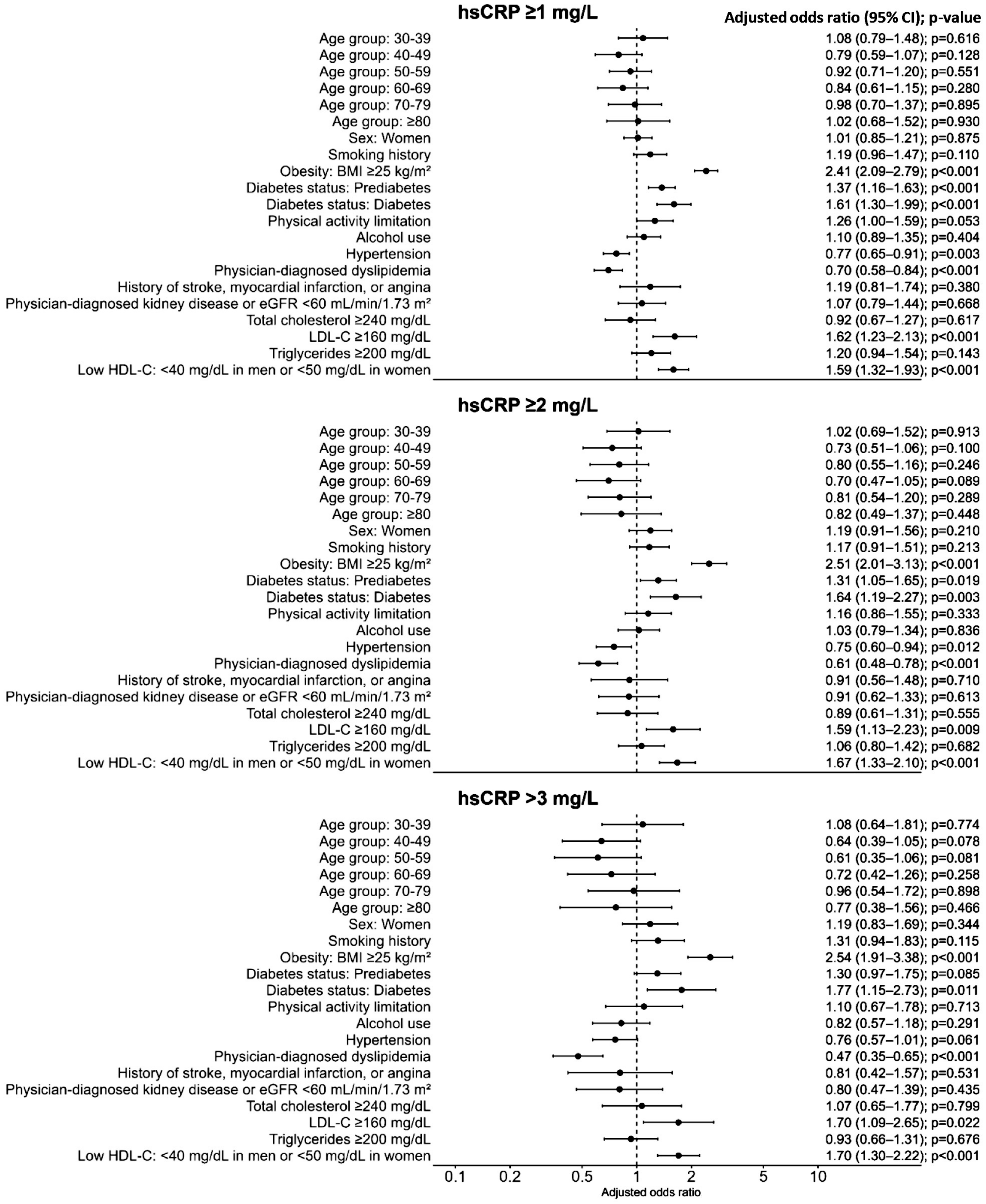

Figure S3. Factors associated with elevated hsCRP levels at different cutoffs in the sensitivity analysis population: survey-weighted multivariable logistic regression analyses. Survey-weighted adjusted ORs and 95% CIs were estimated using multivariable logistic regression models for hsCRP ≥1 mg/L, hsCRP ≥2 mg/L, and hsCRP >3 mg/L. The reference categories were age 20–29 years, men, never-smokers, non-obesity, normal glucose status, no physical activity limitation, no alcohol use, no hypertension, no physician-diagnosed dyslipidemia, no history of cardiovascular disease, no kidney disease, total cholesterol <240 mg/dL, LDL-C <160 mg/dL, triglycerides <200 mg/dL, and HDL-C ≥40 mg/dL in men or ≥50 mg/dL in women. Points indicate adjusted odds ratios, and horizontal lines indicate 95% confidence intervals. Abbreviations: BMI, body mass index; CI, confidence interval; eGFR, estimated glomerular filtration rate; HDL-C, high-density lipoprotein cholesterol; hsCRP, high-sensitivity C-reactive protein; LDL-C, low-density lipoprotein cholesterol.
